# Design of a non-interventional post-marketing study to assess the long-term safety and effectiveness of ocrelizumab in German real world multiple sclerosis cohorts – the CONFIDENCE study protocol

**DOI:** 10.1186/s12883-020-01667-7

**Published:** 2020-03-14

**Authors:** Petra Dirks, Vera Zingler, Jost Leemhuis, Heike Berthold, Stefanie Hieke-Schulz, David Wormser, Tjalf Ziemssen

**Affiliations:** 1 Roche Pharma AG, Emil-Barell-Straße 1, 79639 Grenzach-Wyhlen, Germany; 2 F. Hoffmann-La Roche Ltd, Grenzacherstraße 124, 4070 Basel, Switzerland; 3 Universitätsklinikum Carl Gustav Carus, Zentrum für klinische Neurowissenschaften, Fetscherstr. 74, 01307 Dresden, Germany

**Keywords:** Ocrelizumab, Relapsing multiple sclerosis, Primary progressive multiple sclerosis, Real world data, Non-interventional study, Long-term safety and effectiveness, Disease modifying therapy, MSDS3

## Abstract

**Background:**

Multiple sclerosis (MS) is a chronic disease that requires lifelong treatment. A highly effective drug not only for relapsing but also for progressive forms of MS with a favorable safety profile is needed to further improve overall patient outcomes. Ocrelizumab, a recombinant humanized monoclonal antibody that selectively targets CD20-expressing B-cells, is the first drug indicated for the treatment of adult patients with relapsing forms of MS (RMS) and primary progressive MS (PPMS). Its safety and effectiveness profile has yet to be studied in a large, real-world setting. CONFIDENCE aims to further characterize the safety profile of ocrelizumab in routine clinical practice. In addition, real-world effectiveness data will be collected to complement the efficacy data documented in the pivotal clinical trials.

**Methods:**

CONFIDENCE is a non-interventional, prospective, multicenter, long-term study collecting primary data from 3000 RMS and PPMS patients newly treated with ocrelizumab and 1500 patients newly treated with other selected MS disease-modifying therapies (DMTs). Treatment must be in accordance with the local label and follow routine practice. Data will be collected at approximately 250 neurological centers and practices across Germany. The recruitment period of 30 months started in April 2018. The observation period per patient is planned 7.5 to 10 years, depending on the date of inclusion, regardless of whether patients discontinue treatment. Visits follow routine practice and will be documented approximately every 6 months. The primary endpoint is the incidence and type of uncommon adverse events and death. Statistical analyses will be mainly descriptive and exploratory.

**Discussion:**

CONFIDENCE is a large, non-interventional, post-authorization safety study that assesses long-term safety and effectiveness of ocrelizumab and other DMTs in a real-world setting. Data collected in CONFIDENCE will also be integrated into studies that have been developed to fulfil international regulatory requirements.

## Background

Multiple sclerosis (MS) is a chronic, inflammatory, demyelinating disease of the central nervous system (CNS) [1]. It presents as relapsing (RMS) or primary progressive MS (PPMS). Disease-modifying therapies (DMTs) approved for RMS reduce the number of relapses and slow progression of disease, thus delaying disability in RMS. Ocrelizumab (Ocrevus®) is the only drug approved for PPMS. DMTs modulate the immune system by various mechanisms of action, aiming to reduce the risk of inflammatory disease activity by suppressing peripheral lymphocyte activity affecting or infiltrating the CNS [2]. A concern of medication that targets the immune system is an increase in risk of serious infections and malignancy, especially with prolonged use [3]. MS requires a lifelong therapy; however, long-term use of MS therapies has been associated with the occurrence of serious side effects [4]. Thus, a highly effective drug with a favorable safety profile is still needed to further improve overall patient outcomes.

Ocrelizumab is a recombinant humanized monoclonal immunoglobulin G1 (IgG1) antibody designed to selectively target CD20-positive B cells [5]. In clinical trials, ocrelizumab demonstrated a favorable benefit/risk profile in RMS and PPMS patients [6, 7]. It was approved by the United States (U.S.) Food and Drug Administration (FDA) on 28 March 2017 as first medication for the treatment of adult patients with RMS as well as PPMS, and by the European Medicines Agency (EMA) on 08 January 2018 for treatment of active RMS and early PPMS.

Ocrelizumab demonstrated superior efficacy in a double blind, randomized phase II trial compared with placebo in relapsing-remitting MS (RRMS) (NCT00676715 [8];). Two identical 96-week, randomized, active-controlled phase III trials in RMS patients, OPERA I (NCT01247324) and OPERA II (NCT01412333), demonstrated that ocrelizumab is more efficacious than interferon (IFN) β-1a for the treatment of RMS [6]. A third pivotal double-blind, randomized, placebo-controlled phase III trial in PPMS patients, ORATORIO (NCT01194570), showed that ocrelizumab is more efficacious than placebo for the treatment of PPMS [7]. Results of these trials show that depletion of CD20-positive B cells leads to a significant impact on all measurable parameters of clinical and subclinical disease activity, including relapses, disability progression, and magnetic resonance imaging (MRI) outcomes related to disease progression.

Overall, ocrelizumab showed a favorable safety profile in RMS and PPMS patients. In phase III trials, proportions of adverse events (AEs) and serious AEs were comparable for ocrelizumab and comparator arms [6, 7]. In RMS trials, fewer serious infections were reported for ocrelizumab-treated patients than IFN β-1a-treated patients (1.3% vs. 2.9%); in PPMS trials, serious infections were similar between groups (6.2% [ocrelizumab] and 5.9% [placebo]). Pooled data from phase II trial NCT00676715, OPERA I and II, and ORATORIO implied an imbalance in malignancies in the ocrelizumab treatment group versus pooled IFN β-1a and placebo. The rate of malignancies in ocrelizumab-treated patients, however, remained within the range reported in epidemiological data [6, 7]. Safety data from controlled treatment and open-label extension periods of the phase II and phase III trials are generally consistent with phase IIIb studies CHORDS (NCT0263785), VELOCE (NCT02545868), CASTING (NCT0286101), and OBOE (NCT0268898), which comprise more than 10,000 combined patient years of ocrelizumab exposure [9]. While the pivotal and phase IIIb studies established the safety and efficacy of ocrelizumab under controlled trial conditions in a selected patient population, data are needed to further characterize the safety and effectiveness of ocrelizumab over a long treatment duration and, importantly, in a clinical practice setting.

The non-interventional, post-authorization safety study (PASS) CONFIDENCE will capture long-term safety and effectiveness data of MS patients newly treated with ocrelizumab, i.e. exposed to ocrelizumab for the first time in their treatment history, in a real-world setting. To give this data context, CONFIDENCE will also collect data of MS patients newly treated with selected MS DMTs other than ocrelizumab in parallel. With regard to safety, the study aims to observe general long-term safety with a special focus on uncommon AEs (i.e. AEs with an incidence of 0.1 to 1% [1 to 10 out of 1000 patients] or less; e.g. serious infections and malignancies). In addition, ocrelizumab real-world effectiveness data will be collected over a long duration to complement the efficacy from phase III studies.

Collected safety data will be integrated into global ocrelizumab post-marketing safety studies, which have been developed to fulfil regulatory requirements of various health authorities. CONFIDENCE is thus the central study in the global ocrelizumab post-marketing safety program.

## Methods/design

### Objectives

The primary objective of CONFIDENCE is:

- To assess the long-term safety of ocrelizumab with special focus on the occurrence and characterization of uncommon AEs in patients with MS newly exposed to ocrelizumab.

The key secondary objective is:

- To assess the long-term safety of selected DMTs other than ocrelizumab with special focus on the occurrence and characterization of uncommon AEs in patients with MS exposed to those DMTs for the first time during the course of their MS therapy.

Additional secondary objectives are:

- To estimate long-term effectiveness of ocrelizumab and of other selected DMTs, (no clinical disease activity measured by relapse and disease progression and no treatment discontinuation due to AEs [excluding pregnancies] and lack of effectiveness) in patients with MS newly exposed to ocrelizumab or to other selected DMTs.
- To estimate the incidence of serious infections, malignancies and the malignancy-related mortality rate in patients with MS newly exposed to ocrelizumab or to other selected DMTs.

### Study design

CONFIDENCE is a long-term, prospective, multicenter, non-interventional study that collects primary safety and effectiveness data of patients with RMS and PPMS newly treated with ocrelizumab (ocrelizumab cohort) or patients newly treated with other selected MS DMTs (selected DMT cohort). Data from patients treated with other selected DMTs will be used to present ocrelizumab data in context of real-world MS treatment. These DMTs are alemtuzumab, cladribine, dimethyl fumarate, fingolimod, natalizumab and teriflunomide. In order to avoid the overrepresentation of individual DMTs other than ocrelizumab, an upper limit of 500 patients is defined for each them (see *Study Population*). The decision to treat with ocrelizumab or other DMT must be made by the treating physician before and independently of the decision to enroll the patient in the study. Dosing and treatment duration with ocrelizumab or other DMT will be at the discretion of the physician in accordance with local clinical practice as described in the summary of product characteristics (SmPC) of ocrelizumab (Ocrevus®), alemtuzumab (Lemtrada®), cladribine (Mavenclad®), dimethyl fumarate (Tecfidera®), fingolimod (Gilenya®), natalizumab (Tysabri®) and teriflunomide (Aubagio®). According to routine practice and recommendations by the German Society of Neurology [10] patient visits on which data will be collected are expected every 6 months.

**Fig. 1:**
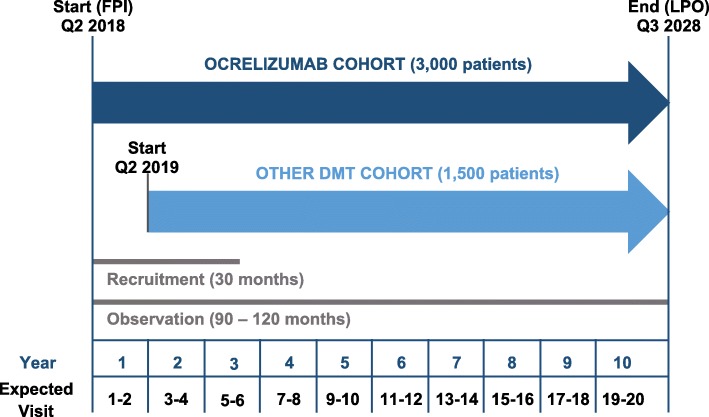
Study design of CONFIDENCE. CONFIDENCE comprises two cohorts, the ocrelizumab cohort and the other DMT cohort. Recruitment into the ocrelizumab cohort started in April 2018, while recruitment into the other DMT cohort will start in Q2 2019. The observation period is planned to continue until the third quarter of 2028. Observation per patient will be 7.5 years (90 months) to 10 years (120 months; in the ocrelizumab cohort) depending on the time of inclusion. Visits follow routine practice and are expected to be documented every 6 months. FPI, first patient in; LPO, last patient out; Q, quarter

The study was registered on 05 March 2018 in the EU PAS Register (http://www.encepp.eu/encepp/studiesDatabase.jsp) under the EU PAS Register Number EUPAS22951. Data will be collected at a planned 250 centers across Germany. The recruitment period will take 30 months. The observation period per patient will continue for 7.5 to 10 years, depending on the date of inclusion. The study started in April 2018 (first record of data on the first study subject in the study database). The end of study (last record of data on last study subject) is estimated for the third quarter of 2028 (Fig. 1). Visits follow routine practice and are planned to be documented approximately every 6 months for up to 10 years. Follow-up visits are planned for the entire study period for all participants, regardless of discontinuation of the studied medication or development of a malignancy.

CONFIDENCE will comply with Good Pharmacoepidemiological Practice [11] guidelines by the International Society of Pharmacoepidemiology (ISPE) and with national and EU requirements for ensuring the well-being and rights of participants in non-interventional PASSs. Ethics votes were obtained in accordance with local legal requirements for non-interventional studies in Germany (professional code of conduct of the medical associations). The independent ethics committee at the Technical University Desden has given the first professional advice for this observational study (Ethikkommission an der Technischen Universität Dresden, Germany; 12 February 2018 and 10 April 2019; reference EK 62022018). Obtaining further ethics votes was the individual responsibility of the participating physicians.

### Study population

Data will be collected from 3000 patients with RMS or PPMS newly treated with ocrelizumab (ocrelizumab cohort; Fig. 1) and 1500 patients newly treated with selected MS DMTs other than ocrelizumab. Recruitment for both cohorts will take place at the same centers. To avoid over-representation of individual DMTs, a maximum of 500 patients will be included for each DMT other than ocrelizumab. This will be achieved by regularly monitoring the recruitment status and stopping enrolment of patients who receive the respective DMT when the maximum is reached.

Data from CONFIDENCE will be used in global post-marketing safety commitments, which, among other objectives, aim to investigate the occurrence of malignancies, particularly breast cancer, during ocrelizumab therapy. In order to adequately power breast cancer event rate analyses, at least 70% of each cohort will be female. This will be ensured by monitoring the recruitment status and stopping enrollment of male patients when their quota is reached.

Inclusion criteria include signed informed consent, diagnosis of MS, age ≥ 18 years, and patients must be newly treated (for the first time during the course of their MS therapy) with ocrelizumab (for the ocrelizumab cohort) or the specific other DMTs (for the other DMT cohort) (up to 30 days prior to or 60 days after study enrollment) according to the local label irrespective of the reason for starting the treatment. Exclusion criteria include participation in an interventional study investigating MS DMTs, patients who have received ocrelizumab or their new MS DMT other than ocrelizumab more than 30 days prior to study entry, and patients who have been previously treated with rituximab. Patients who are included in the ‘other DMT cohort’ must not have previously received ocrelizumab therapy.

### Endpoints

All CONFIDENCE endpoints are listed in Table 1.

**Table 1:** Safety, effectiveness and exploratory endpoints of CONFIDENCE

**Primary safety endpoint (ocrelizumab cohort)**	Incidence and type of uncommon AEs (including AEs with an incidence of 0.1 to 1% [1 to 10 out of 1000 patients] or less) and death (including primary and underlying causes) in patients with MS newly exposed to ocrelizumab
**Key secondary safety endpoint (other DMT cohort)**	Incidence and type of uncommon AEs (including AEs with an incidence of 0.1 to 1% [1 to 10 out of 1000 patients] or less) and death (including primary and underlying causes) in patients with MS newly exposed to selected DMTs other than ocrelizumab
**Secondary effectiveness endpoints (both cohorts)**	Treatment success: proportion of patients with no clinical disease activity measured by relapse and disease progression and no discontinuation of current treatment due to AEs (excluding pregnancies) and lack of effectiveness
	Annualized relapse rate since start of treatment
	Proportion of patients with relapses since start of treatment
	Mean number of relapses per patients within the last 3 years before start of treatment
	Change in expanded disability status scale (EDSS) from baseline
	Proportion of patients with confirmed disability progression (CDP)
	Time to onset of CDP
	Time to onset of confirmed disability improvement (CDI)
	Patient reported outcomes: treatment satisfaction, cognitive function, and health related quality of life
**Other safety endpoints (both cohorts)**	Incidence of all AEs
	Incidence of serious infections
	Incidence of malignancies
	Incidence of mortality due to malignancies
**Exploratory endpoints (both cohorts)**	Mean number of gadolinium (Gd) enhancing T1 lesions as detected by brain magnetic resonance imaging (MRI)
	Change from baseline in MRI lesions (T2 and Gd + lesions)
	Global impression on the disease course as reported by physician and patient using an adapted ‘Clinical Global Impression (CGI)’ scale: Severity Score (change from baseline) and Improvement Score (by descriptive statistics [mean, standard deviation])
	Change from baseline in pharmacoeconomic outcomes as measured by ‘Work Productivity and Activity Impairment Questionnaire: Multiple Sclerosis (WPAI:MS)’

### Data collection

Data collection according to standard of care starts after written informed consent by the patient and can take place before the first administration of ocrelizumab or other DMT (up to 30 days before or 60 days after study enrollment). Only available data will be collected; no additional diagnostic or monitoring procedures shall be applied to the patients outside of routine clinical practice. Data will be entered online by study site staff into electronic case report forms (eCRFs) based on the MS management system 3D (MSDS3D; MedicalSyn, Stuttgart, Germany) adapted for CONFIDENCE. This software had previously been developed to collect real-world safety data [12–14] and is well established as an eCRF tool in MS-related observational studies [15–18].

Data will be collected at screening, baseline, follow-up visits, and upon study completion/withdrawal as summarized in Table 2. As this is a non-interventional study, data are only documented if they are generated according to clinical routine; no examinations are carried out specifically for this study. There are no mandated study visits, and data from any encounter with the neurologist during follow-up will be entered (anticipated to occur every ~ 6 months). Follow-up is planned regardless of whether patients discontinue their current MS treatment.

**Table 2:** Data collection overview

Data to be collected	Study Enrollment (Screening)	Baseline (Study Entry)	Data Collection (approximately every ~ 6 months)	Study Completion/ Study Withdrawal
Patient demographics	X			
Informed consent	X			
Inclusion/exclusion criteria	X			
Vital signs and measurements^a^		X	X	X
Pregnancy status (patient-reported)		X	X	X
Pregnancies		whenever they occur		
Laboratory test results^b^		X	X	X
John Cunningham Virus (JCV) antibody status / index		X		
MS disease history^c^		X		
MS treatment history^d^		X		
Medical history and comorbidities		X		
Clinically significant medical and surgical history (including previous malignancies and precancerous lesions)		X		
Malignancy risk factor information^e^		X		
Cancer screening^f^		X	X	X
MS symptoms^g^		X	X	X
Current MS DMT administration information^h^		X	X	X
Patient Reported Outcomes (PROs): Treatment Satisfaction Questionnaire for Medication (TSQM) 1.4, Multiple Sclerosis Impact Scale (MSIS)-29v2, adapted Clinical Global Impression (CGI), Symbol Digit Modality Test (SDMT)		X	X	X
Pharmacoeconomic outcomes: Work Productivity and Activity Impairment Questionnaire: MS (WPAI:MS)		X	X	X
Premature termination incl. Reasons for study withdrawal				X
Continuation of therapy or reasons for treatment discontinuation			X	X
Prior and concomitant medications other than MS medication (up to 3 months prior to study entry)		X	X	X
Any malignancies^i^		whenever they occur		
All serious and non-serious AEs incl. Non-melanoma skin cancer (NMSCs) and information on reasons for death		whenever they occur		
Death information			X	X

^a^ Height, weight, blood pressure, heart rate and temperature

^b^ Blood cell count, immunoglobulins, liver enzymes, renal status, clinical chemistry, flow cytometry. Viral serology (only at baseline)

^c^ Including MS date of diagnosis, type of MS, duration of MS, MS disease symptom history, relapse history, EDSS (or proxy) and MRI results (if available)

^d^ Prior use and duration of therapies for MS and prior use and duration of immunomodulatory, immunosuppressive, and anti-neoplastic agents (if any)

^e^ Including tobacco use history, alcohol use history, family disease history, genetic testing such as BRCA1 and 2, personal history of malignancy, or other risk factors including reproductive history (women and undifferentiated only); the selection of these risk factors is based on the VERISMO study (for further details on VERISMO, see Discussion)

^f^ Including gynecological consultation, breast check, dermatological check, or other malignancy/cancer screening tests and procedures (e.g., mammography, Pap test, colonoscopy, laboratory malignancy markers)

^g^ Including MS relapse during treatment period, MS type changes, date of last administration of Ocrelizumab (if applicable), MS DMT changes and rationale, most recent EDSS score since last encounter (if available), most recent MRI results since last encounter (if available)

^h^ Dates of administration (start, stop, and restart dates), dose, dosing frequency, reason for discontinuation (if applicable)

^i^ Medical records regarding in-depth malignancy information will be solicited from the patient’s oncologist or pathologist (only collected if a malignancy is identified)

Data sources include patient charts, interviews, medical examinations and questionnaires on patient reported outcomes (PROs; see below). The completion of such questionnaires is not routine practice but is allowed according to local regulations (published by a joint notification of the two German higher federal authorities [19];). Available clinical information on new malignancies, defined as the first occurrence of a cancer diagnosis during study follow-up including recurrences, will be provided by the treating physicians (treating neurologist and oncologist/pathologist). In addition data linkage with national and regional cancer registries will be explored to ensure identification of all malignancy cases in the study population.

This study uses five patient questionnaires to obtain data on PROs and on pharmacoeconomic outcomes: the Treatment Satisfaction Questionnaire for Medication (TSQM) 1.4 [20], the Multiple Sclerosis Impact Scale (MSIS)-29v2 [21], an adapted version (with a six-point improvement scale) of the Clinical Global Impression (CGI) [22], the Symbol Digit Modality Test (SDMT) [23], and the Work Productivity and Activity Impairment Questionnaire: Multiple Sclerosis (WPAI:MS) [24].

### Statistical analysis

The sample size is planned to allow for the detection of AEs with an incidence of at least 0.1% (1 out of 1000 patients) at least once with a probability of 95%. Applying the Poisson distribution model for uncommon events, the total sample size results in approximately 3000 patients – the size of the ocrelizumab cohort. Taking into account that ocrelizumab is currently the only approved disease modifying medication for PPMS and the resulting unmet need in this population, approximately 700–1000 patients in the ocrelizumab cohort are expected to be PPMS patients. The other DMT cohort of 1500 patients will allow for the detection of AEs with an incidence rate of at least 0.2% (1 out of 500 patients) at least once with a probability of 95%.

Statistical analyses will be mainly descriptive and exploratory in nature. Statistical modeling and inferential statistics will also be used, but will be interpreted in an exploratory fashion. Missing data will not be substituted (‘observed cases analysis’). Three analysis sets will be used: the Enrolled Set (ES; all patients recorded in the clinical database who have given informed consent), the Safety Set (SS; all patients in the ES treated with at least one dose of the studied medicinal product documented in the eCRF), and the Full Analysis Set (FAS; all patients of the SS who had at least one documentation after start of therapy). Based on patients‘potential switch of treatment, four subsets will be derived from the SS and FAS set separately. These are: patients who receive ocrelizumab during the whole course of the study plus patients who switch from ocrelizumab to DMT until this treatment switch (subset 1), patients who receive DMT during the whole course of the study plus patients who switch from DMT to ocrelizumab until this treatment switch (subset 2), patients who switch from DMT to ocrelizumab, after treatment switch (subset 3), and patients who switch from ocrelizumab to DMT, after treatment switch (subset 4).

All effectiveness analyses will be performed separately for each combination of cohort (ocrelizumab and other MS DMTs), MS type (RMS and PPMS) and FAS subset 1 to 4. Similarly, all safety analyses will be performed separately for each combination of cohort (ocrelizumab and other MS DMTs), MS type (RMS, PPMS, and total) and SS subset 1 to 4. Subgroup analyses will comprise EDSS at baseline (0–3.5 / ≥4 for RMS, 0–5.5 / > 5.5–6.5 /≥7 for PPMS), MS-specific pre-treatment, age (< 40 / ≥40 years for RMS, ≤40 / 41–55 / > 55 years for PPMS), sex (male vs. female).

For continuous data, the mean, standard deviation, median, range (minimum, maximum), and interquartile range (Q1, Q3) will be calculated. Categorical data will be displayed by absolute and relative frequencies. Incident rates will be calculated for safety endpoints. Time-to-event endpoints will be analyzed using Kaplan-Meier product limit methods to estimate the survival distribution curves and the median survival time. A negative binomial model will investigate the annualized relapse rates. The number/percentage of patients with a change in MS / RMS type at any time during the study will be summarized and the time to change in years will be calculated. The number/percentage of patients with relapses any time and per visit during study will be calculated. Methods of survival analysis (Kaplan-Meier summary and corresponding Kaplan-Meier plot) will be performed. These also will be repeated for all subgroups. A logistic regression model will be used to evaluate in how far the probability for treatment success is influenced by covariates like EDSS baseline score, age (continuous), sex, categories based on enrolment date, MS type. The full model is specified in the statistical analysis plan. Treatment exposure, the number of administrations per patient and the total dose per patient will be summarized as numeric variables. The analysis on treatment exposure will be repeated for all subgroups. Annual interim analyses are planned starting when 1-year data of at least 500 patients are available.

## Discussion

### Strengths of this study

Data collected from the pivotal randomized clinical trials (RCTs) provide a sound body of evidence for the efficacy and safety of ocrelizumab treatment for RMS and PPMS [6, 7] and led to the approval of ocrelizumab. CONFIDENCE is a large, non-interventional study with less restrictive enrolment criteria, greater patient number and longer observation periods than the pivotal studies. The real-world evidence obtained in CONFIDENCE will help answer questions that cannot be answered by RCTs, question relevant for daily practice, the medical community and health insurance [25].

CONFIDENCE offers the possibility to detect rare safety events in enrolled patients regardless of a change in treatment or discontinuation of ocrelizumab. The comprehensive collection of risk factors and an adjudication process for newly occurred malignancies facilitates the reliable interpretation of malignancy events. This will be a solid basis for the investigation of a possible effect of treatment on the occurrence of cancer. Furthermore, this study will help learn more about the treatment algorithm of MS patients, because it collects data on underlying reasons for therapy changes and discontinuations.

### Minimization of limitations

Rather than observing ocrelizumab in isolation, the study aims to present ocrelizumab data in the context of the safety profiles of other selected approved MS DMTs. Consequently, a cohort of patients newly treated during the course of their MS therapy with alemtuzumab, cladribine, dimethyl fumarate, fingolimod, natalizumab, or teriflunomide will be observed in parallel with the ocrelizumab cohort. However, label differences between ocrelizumab and other DMTs are potential confounding factors. In order to be able to minimize such potential confounding factors and to take different prerequisites for individual drug therapies into account, factors associated with treatment choice (e.g. history of relapse and MRI activity as well as the most recent EDSS) will be collected at study enrollment.

To address potential enrollment bias between cohorts, sites are encouraged to enroll patients into both treatment cohorts and to include patients representing the site’s general population regarding patient disposition and treatment regimen. Patients will be enrolled as new users at the time of ocrelizumab or other MS DMT treatment initiation; this will reduce potential bias (healthy user bias, depletion of susceptibles) associated with the study of long-term medication users. This will also ensure that the same starting point applies to each drug treatment and that all side effects can be recorded including those that arise from at least one dose. To reduce possible misclassification, all sites will undergo standardized training and utilize standardized documentation for the completion of eCRFs at enrollment and for each follow-up assessment, specifically on collecting exposure and outcome variable information.

The willingness to introduce new drugs such as ocrelizumab into MS therapy is difficult to predict and may affect the achievement of recruitment targets. Continuous monitoring of patient enrollment at the site and country level will allow a rapid response to under-recruiting, e.g. by initiation of additional sites.

### CONFIDENCE as a central element of the global ocrelizumab post-marketing safety program

**Fig. 2:**
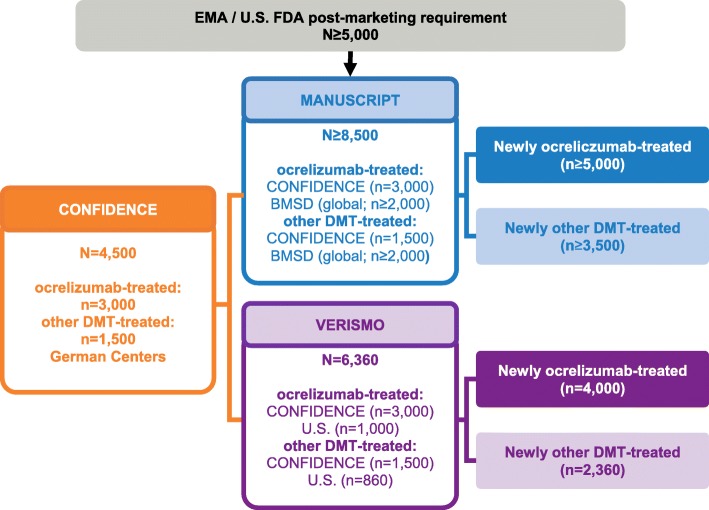
Integration of safety data from CONFIDENCE into other global post-marketing safety studies. Safety data from the German study CONFIDENCE will be integrated into the global post-marketing safety studies MANUSCRIPT and VERISMO. MANUSCRIPT (blue) will combine data from CONFIDENCE (orange) and from multiple existing international MS and population registries (Big MS Data Group, BMSD, which combines clinical data from multiple registries, i.e. MSBase, France OFSEP [Observatoire Français de la Sclérose en Plaques], Denmark, Sweden and Italy). VERISMO (purple) will combine prospective data collected at sites in the United States (U.S.) and the German CONFIDENCE sites

In order to fulfil regulatory requirements of the FDA and the EMA, a program comprising two observational studies – VERISMO (EUPAS30752) and MANUSCRIPT (EUPAS28619) – has been developed to characterize the long-term safety of ocrelizumab in the post-marketing setting. VERISMO and MANUSCRIPT are multi-source, international, non-interventional, longitudinal cohort post-marketing safety studies involving patients with MS who have newly initiated treatment with ocrelizumab and other MS DMTs [26]. VERISMO aims to determine the incidence rate of breast cancer and all malignancies, is expected to enroll a total of 6360 patients (including 4000 patients treated with ocrelizumab; Fig. 2), and collects prospective data at study sites in the US and Germany. MANUSCRIPT aims to estimate rates of serious AEs, including malignancies and serious infection. It is expected to collect data from at least 8500 MS patients (including ≥5000 patients treated with ocrelizumab; Fig. 2), and is primarily based on data from multiple existing international MS and population registries (i.e. Big MS Data Group, BMSD).

CONFIDENCE is aligned with the FDA and EMA commitments, and safety data collected in CONFIDENCE will be integrated in VERISMO and MANUSCRIPT. Therefore, CONFIDENCE is the central study in the global ocrelizumab post-marketing safety program (Fig. 2). All pregnancies observed within CONFIDENCE will be additionally transferred to the German nationwide Multiple Sclerosis and Pregnancy Registry (Ruhr-University Bochum) and the Ocrevus Pregnancy Registry (EU PAS register number not available yet) if the pregnant woman has given her informed consent. In contrast to VERISMO and MANUSCRIPT, CONFIDENCE also investigates a number of effectiveness endpoints (Table 1), which distinguishes it from the other two studies.

## Data Availability

Qualified researchers may request access to individual patient level data through the clinical study data request platform (www.clinicalstudydatarequest.com). Further details on Roche’s criteria for eligible studies are available here (https://clinicalstudydatarequest.com/Study-Sponsors/Study-Sponsors-Roche.aspx). For further details on Roche’s Global Policy on the Sharing of Clinical Information and how to request access to related clinical study documents, see here (https://www.roche.com/research_and_development/who_we_are_how_we_work/clinical_trials/our_commitment_to_data_sharing.htm).

## Abbreviations

AE: Adverse event
BMSD: Big MS Data Group
CDI: Confirmed disability improvement
CDP: Confirmed disability progression
CGI: Clinical Global Impression
CNS: Central nervous system
DMT: Disease modifying therapy
eCRF: Electronic Case report form
EDSS: Expanded disability status scale
EMA: European Medicines Agency
FDA: Food and Drug Administration
FPI: First patient in
Gd: Gadolinium
GPP: Good Pharmacoepidemiological Practice
IFN: Interferon
IgG1: Immnunoglobulin G1
ISPE: International Society of Pharmacoepidemiology
JCV: John Cunningham Virus
LPO: Last patient out
MRI: Magnetic resonance imaging
MS: Multiple sclerosis
MSDS3D: Multiple sclerosis management system 3D
MSIS: Multiple Sclerosis Impact Scale
NMSC: Non-melanoma skin cancer
OFSEP: Observatoire Français de la Sclérose en Plaques
PASS: Post-authorization safety study
PPMS: Primary progressive multiple sclerosis
PRO: Patient reported outcome
RMS: Relapsing multiple sclerosis
RRMS: Relapsing-remitting Multiple Sclerosis
SDMT: Symbol Digit Modality Test
SmPC: Summary of Product Characteristics
TSQM: Treatment Satisfaction Questionnaire for Medication
U.S.: United States
WPAI:MS: Work Productivity and Activity Impairment Questionnaire: Multiple Sclerosis
